# Influence of the first radioactive iodine ablation on peripheral complete blood count in patients with differentiated thyroid cancer

**DOI:** 10.1097/MD.0000000000004451

**Published:** 2016-09-02

**Authors:** Tianpeng Hu, Zhaowei Meng, Guizhi Zhang, Qiang Jia, Jian Tan, Wei Zheng, Renfei Wang, Xue Li, Na Liu, Pingping Zhou, Arun Upadhyaya

**Affiliations:** Department of Nuclear Medicine, Tianjin Medical University General Hospital, Tianjin, P.R. China.

**Keywords:** differentiated thyroid cancer, hemoglobin, platelet, radioactive iodine, red blood cell, white blood cell

## Abstract

Radioactive iodine (RAI) is considered to be related with hematologic changes. This study aimed to evaluate influence of the first RAI ablation on peripheral complete blood count (CBC) in patients with differentiated thyroid cancer (DTC).

Data of CBC at baseline and 6 months after RAI were obtained in 385 patients with DTC with approximately 3700 MBq ^131^I (ranging 2220–7585 MBq). Further comparison was done in 196 patients with 1-month postablation data available. Routine blood examinations were performed to determine impact of RAI on white blood cell (WBC), red blood cell (RBC), hemoglobin, platelet, neutrophil, lymphocyte, and monocyte in both sexes. Continuous variables were compared by paired *t* tests and independent samples *t* test, and categorical variables were compared by chi-square analysis. Data with repeated measurements were analyzed by analysis of variance.

The first RAI after thyroidectomy was associated with mild, yet significant declines in WBC, platelet, and lymphocyte, which persisted for 6 months. One month after RAI, significant declines were found in all CBC, including RBC and hemoglobin (all *P* < 0.05). While CBC partly recovered 6 months after RAI, this follow-up CBC still demonstrated significant declines in WBC, platelet, and lymphocyte (all *P* < 0.05) without gender differences. Significant rises in RBC and hemoglobin in males and females were found. The decline of platelet in females was more obvious than in males at 3700 to 4440 MBq of RAI. On the contrary, the rises of RBC and hemoglobin in males were higher than in females. There were no significant complications during the follow-up.

WBC and platelet decreased obviously 1 month after RAI. While they partly recovered 6 months after RAI, they were still lower than the baseline. However, RBC and hemoglobin transiently decreased at 1 month and then increased to levels even higher than baseline 6 months later. At 3700 to 4440 MBq of RAI, the decline of platelet in females was more obvious than in males. Yet, rises of RBC and hemoglobin in males were higher than in females. The risks associated with these changes are unlikely to outweigh the potential benefits of RAI in patients with DTC.

## 1 Introduction

Differentiated thyroid cancer (DTC) is the most common endocrine malignancy and its incidence is increasing.^[1]^ Radioactive iodine (RAI) is a commonly used therapeutic modality in patients with DTC after thyroidectomy, either for ablation of the postsurgical thyroid remnant or for treatment of recurrent and metastatic disease.^[2]^ While high, cumulative administered activities have been associated with an increased risk of secondary malignancies,^[3–5]^ life-threatening long-term complications from repeated RAI are rarely seen. However, side effects including salivary gland dysfunction,^[6,7]^ nasolacrimal obstruction,^[6–9]^ reproductive disturbances, conjunctivitis,^[6,8]^ and hematologic abnormalities^[10,11]^ are not infrequently observed in the first several months after RAI ablation. Fortunately, majority of these early side effects are often transient and barely have long-term clinical significance.

Despite anecdotal evidence of occasional bone marrow suppression after a single treatment of RAI,^[7]^ the risk of anemia, leukopenia, and thrombopenia 6 months after an initial ablative treatment of RAI is not well defined. Therefore, we evaluated the potential impact of RAI after traditional thyroid hormone withdrawal (THW) on the complete blood count (CBC) in 6-month period, with emphasized focus on gender and age.

## 2 Methods

### 2.1 Patients

In this retrospective review, we evaluated a consecutive series of 385 patients with DTC, from the year 2012 to 2015, undergoing initial RAI remnant ablation (after total thyroidectomy) in whom a CBC was available in the medical record both at the time of ablation (1–3 months after surgery) and at the time of the first follow-up whole-body scan approximately 6 months later. Patients were excluded from the study if they were taking any medications known to affect CBC, if they received a second dose of RAI within 6 months, if they were known to have any baseline hematologic diseases or any abnormalities in CBC prior to initial RAI therapy, or if they received external beam radiation therapy and/or chemotherapy before RAI ablation or within 6 months. In addition, 197 of 385 patients with DTC also had CBC records 1 month after RAI therapy. This study received approval from our Institutional Ethical Review Board.

### 2.2 Protocol

All RAI remnant ablation was performed after THW preparation. In brief, all patients were instructed to follow a low iodine diet before, during, and 1 month after RAI therapy. Patients were taken off levo-thyroxine 3 to 4 weeks before RAI therapy. RAI dose was administered orally. Patients were encouraged to drink more water after ablation therapy and to empty bladder as soon as possible. Post-therapy whole-body scan was performed 3 to 5 days later. Whole-body radioactivity after RAI ablation was monitored at least once a day by using a hand-held portable ionchamber survey meter at a distance of 1 m from the middle of the neck. Patients were hospitalized for a period of 4 to 5 days and were advised to sleep separately for a period of 7 days and to limit close contact with relatives for the same period. The administered activity was selected based on the findings of clinicopathological features, the extent of regional and distant metastases, diagnostic whole-body scans, age, renal as well as cardiac function of each individual case.^[12]^ An automated CBC was obtained <3 days before RAI administration. Repeated RAI or diagnostic scan was taken approximately 6 months after the initial RAI therapy when hematologic parameters were re-examined.

### 2.3 Parameter measurement

All serum blood tests were performed in the hematology laboratory at Tianjin Medical University General Hospital using an automated CBC analyzer (Sysmex xn-2000, Sysmex Corporation, Kobe, Japan). The normal range of hemoglobin (Hb) was 130 to 175 and 115 to 150 g/L for males and females, respectively, and red blood cell (RBC) 4.3 to 5.8 × 10^12^/L and 3.8 to 5.1 × 10^12^/L for males and females, respectively. The normal range of white blood cell (WBC), platelet, neutrophil, and lymphocyte were 3.5 to 9.5 × 10^9^/L, 125 to 350 × 10^9^/L, 1.8 to 6.3 × 10^9^/L, and 1.1 to 3.2 × 10^9^/L for both genders, respectively.

### 2.4 Statistical analysis

Data were generally presented as the mean ± standard deviation (SD). Continuous variables were compared by using paired *t* tests and independent samples *t* test, and categorical variables were compared by using chi-square analysis. Data with repeated measurements were analyzed by using analysis of variance. A *P* value of <0.05 was considered statistically significant. All statistical analyses were performed by using Statistical Product and Service Solutions version 21.0 for Windows.

## 3 Results

### 3.1 Clinical characteristics

There were 385 patients with DTC, who had a CBC available for review both before the first RAI ablation and 5.45 ± 1.19 months (range 3–9 months) after the first RAI ablation (Table 1). The majority were females (73.50%) with a mean age of 47.36 years. All had undergone total thyroidectomy for predominantly papillary thyroid cancer (90.91%) and follicular variant of papillary thyroid cancer (8.05%). The median administered RAI activity was 3811 MBq (range 2220–7585 MBq).

**Table 1:**
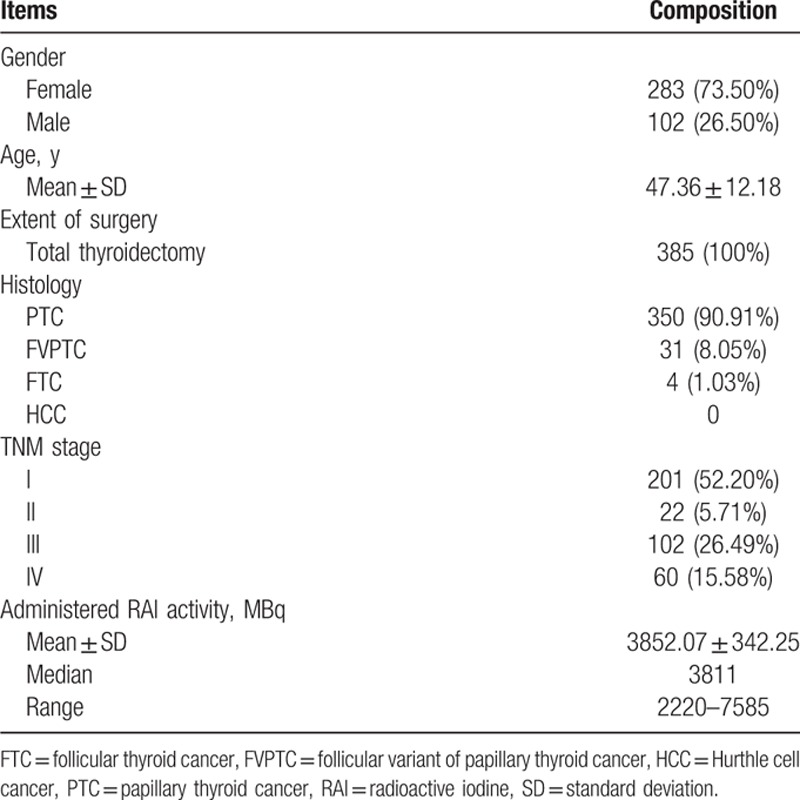
Baseline characteristics of the patients.

### 3.2 CBC findings in the full cohort

Six months after the first RAI therapy, 4.16% of patients (n = 16) exhibited abnormal WBC, 12.99% (n = 50) abnormal Hb, 11.17% (n = 43) abnormal RBC, 5.19% (n = 20) abnormal platelet, 5.97% (n = 23) abnormal neutrophil, and 6.75% (n = 26) abnormal lymphocyte.

Results of CBC findings before and after RAI were shown in Table 2. Even though the mean remained within the normal reference range during follow-up, significant declines in WBC, platelet, and lymphocyte were seen 6 months after RAI ablation. On the contrary, significant increases were seen in RBC and Hb. No significant changes in neutrophil and monocyte were detected. At the 6-month re-evaluation point, no patient suffered severe hematologic complications. None had thrombopenia (lowest platelet count of 113 × 10^9^/L). One patient had leukopenia (WBC of 2.8 × 10^9^/L) and 3 female patients had anemia (Hb < 100 g/L). The patient who had leukopenia was requested to take oral Leucogen tablets (20 mg tid). The 3 patients who had anemia (Hb < 100 g/L) were requested to take oral Shengxuening tablets (0.1 g tid). The main ingredients are 2-thiazolidineacetic acid, 4-carboxy-alpha-phenyl-, alpha-ethyl ester for Leucogen and sodium iron chlorophyllin for Shengxuening, respectively. They recovered rapidly after oral medicine therapy. Compared with the preablation data, the follow-up data done 6 months later revealed that WBC count was lower in 62.08% of the patients, platelet count lower in 55.06% of the patients, yet Hb lower in 29.09% of the patients.

**Table 2:**
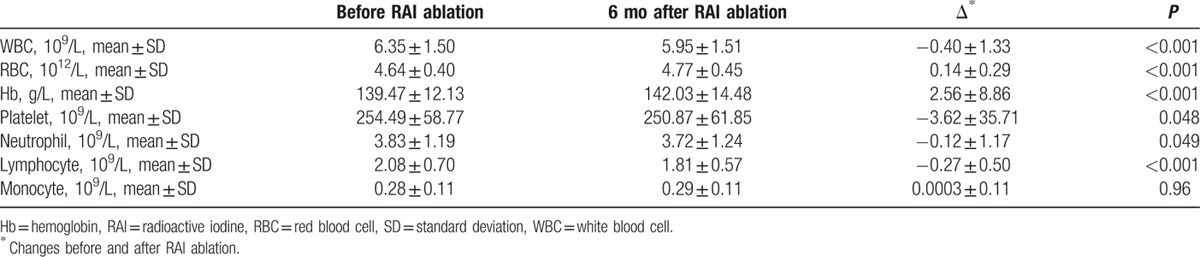
Complete blood cell changes before and after RAI ablation for all patients.

### 3.3 CBC findings analyzed by gender

Gender appeared to have little effect on the baseline CBC as no significant differences were detected in the baseline hematologic parameters. There were no significant differences on WBC, platelet, neutrophil, lymphocyte, and monocyte changes between male and female when comparing the baseline data with those obtained at 6-month follow-up (Table 3). Significant differences were found on RBC and Hb changes between males and females. These changes in males were more obvious than in females (0.18 ± 0.28 vs 0.12 ± 0.29 × 10^12^/L for RBC, *P* = 0.049; 4.71 ± 7.41 vs 1.79 ± 9.21 g/L for Hb, *P* = 0.004).

**Table 3:**
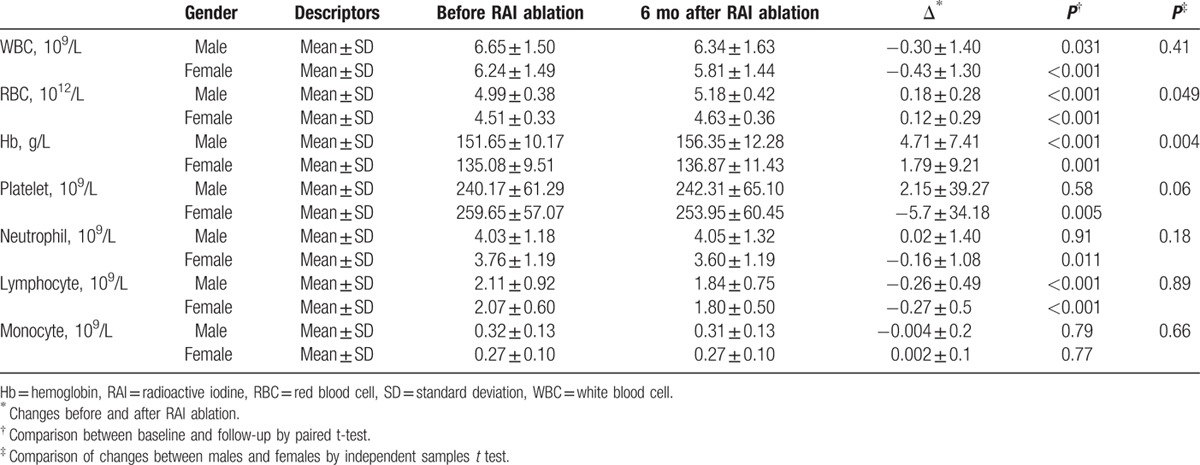
Complete blood cell changes before and after RAI ablation according to gender.

### 3.4 CBC findings analyzed by administered RAI activity

Over the range of different administered activities in this study, no significant dose–response relationship was detected in CBC between baseline and follow-up (Table 4). RAI activity grouping was based on the empiric dosing categories according to our experience: <3700, 3700 to 4440, and more than 4440 MBq. In terms of platelet, a significant difference of the changed value was found between the <3700 MBq group and more than 4440 MBq group by post-hoc test (12.88 ± 33.40 vs −12.68 ± 39.64, *P* = 0.032). When data were analyzed by different genders (Table 5), significant differences in Hb and platelet were seen at 3700 to 4440 MBq levels of administered activities (both *P* = 0.02). By multiple comparison, significant differences were detected in RBC, Hb, and platelet between males and females when we compared CBC at baseline with follow-up. In specific, in 3700 to 4440 MBq group, rising changes in males were more obvious than in females for RBC and Hb (0.19 ± 0.28 vs 0.11 ± 0.29 × 10^12^/L, *P* = 0.04 for RBC; 4.81 ± 7.41 vs 1.41 ± 9.18 g/L, *P* = 0.002 for Hb). While significant rise was found in the platelet count for male, significant decline was detected for female (4.07 ± 40.17 vs −6.75 ± 33.26 × 10^9^/L, *P* = 0.01).

**Table 4:**
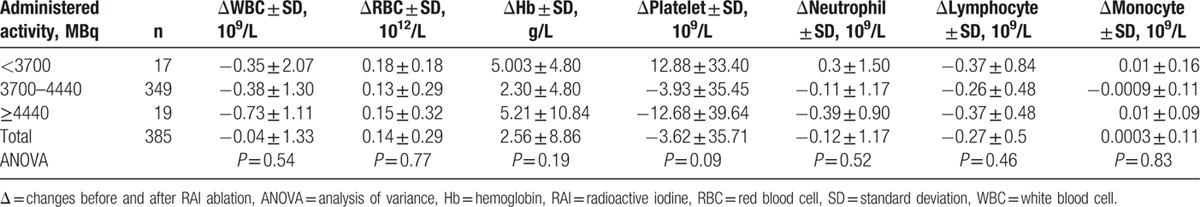
Mean changes in blood cells (6 months after RAI treatment from baseline) according to administered activity of RAI.

**Table 5:**
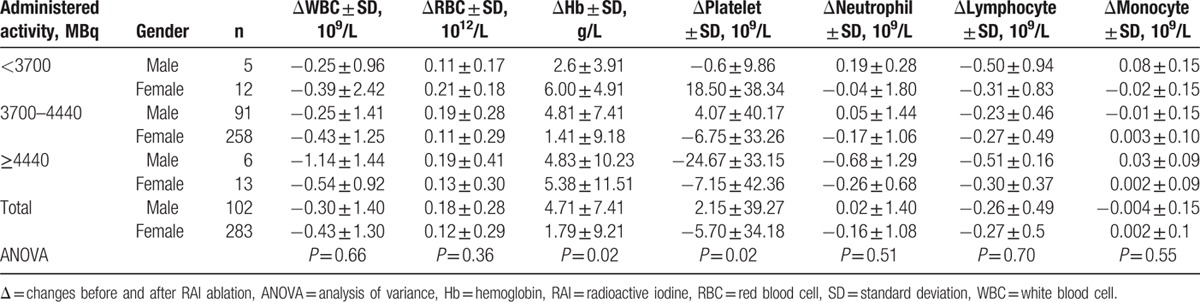
Mean blood cell changes 6 months after the first RAI treatment for male and female according to RAI dose.

In addition, there was no significant difference in the administered RAI activities received by those patients who developed leukopenia at the 6-month follow-up (n = 7, 3930 ± 299 MBq) compared with those patients in whom WBC remained within the normal range (n = 369, 3854 ± 341 MBq). There was no significant difference in administered RAI activities given to those who developed erythropenia in 6 months (n = 5, 3700 ± 454 MBq) compared with those who maintained a normal RBC range (n = 359, 3858 ± 353 MBq). Likewise, there were no significant differences for thrombopenia group (n = 2, 4015 ± 78 MBq) versus normal platelet group (n = 365, 3847 ± 334 MBq), and for anemia group (n = 13, 3905 ± 230 MBq) versus normal Hb group (n = 335, 3845 ± 354 MBq).

### 3.5 CBC findings analyzed by age

As shown in Table 6, we did not detect significant CBC changes with increased age groups. Similar results were demonstrated if data were analyzed by male and female separately (Table 7). By multiple comparison, significant difference was found between male and female in 50 to 60 years group for platelet (12.00 ± 47.47 vs −3.26 ± 30.82 × 10^9^/L, *P* = 0.04). In 40 to 50 years group, significant differences were detected in RBC and Hb between male and female (0.28 ± 0.30 vs 0.11 ± 0.29 × 10^12^/L, *P* = 0.02 for RBC; 6.84 ± 8.38 vs 1.86 ± 7.23 g/L, *P* = 0.02 for Hb).

**Table 6:**
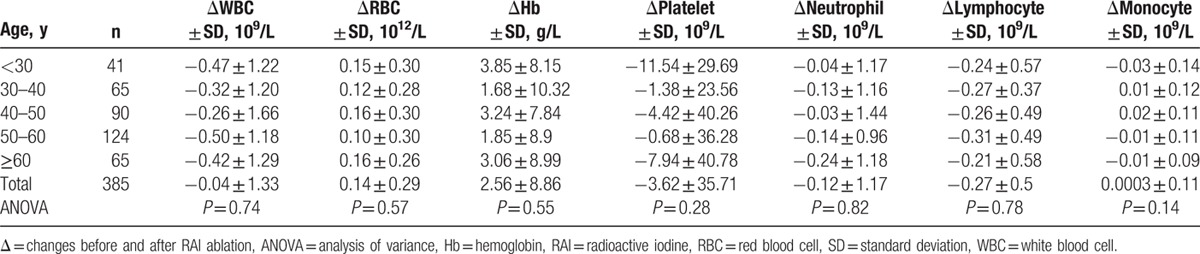
Mean changes in blood cell changes (6 months after RAI treatment from baseline) according to patients’ age.

**Table 7:**
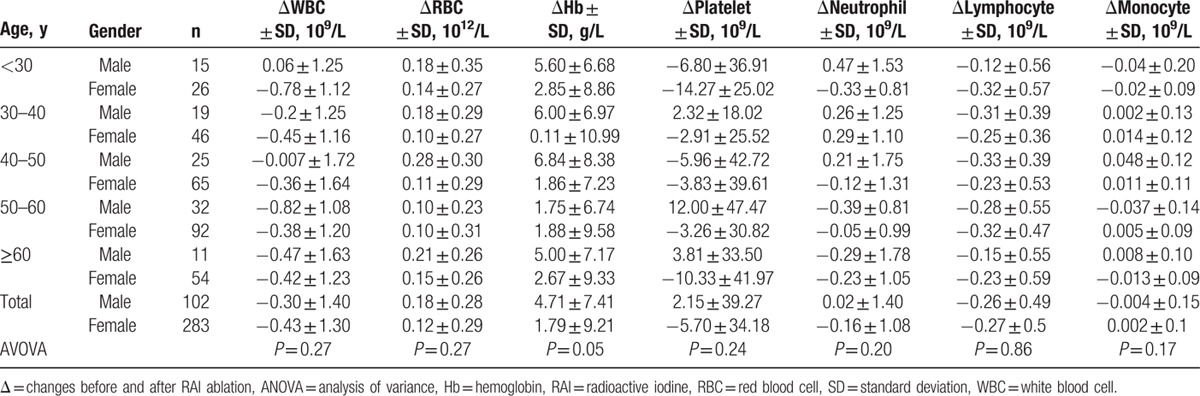
Mean blood cell changes 6 months after the first RAI treatment for male and female according to patients’ age.

Besides, the CBC values reached a nadir at 1-month post-RAI therapy (Table 8). For example, the decrease of WBC from baseline value was 12.69% and 7.11% at 1 and 6 months after RAI therapy (both *P* < 0.001). Interestingly, significant declines were presented in RBC and Hb 1 month after the first RAI treatment. However, 6 months later, significant rises were found in RBC and Hb, even higher than baseline values.

**Table 8:**
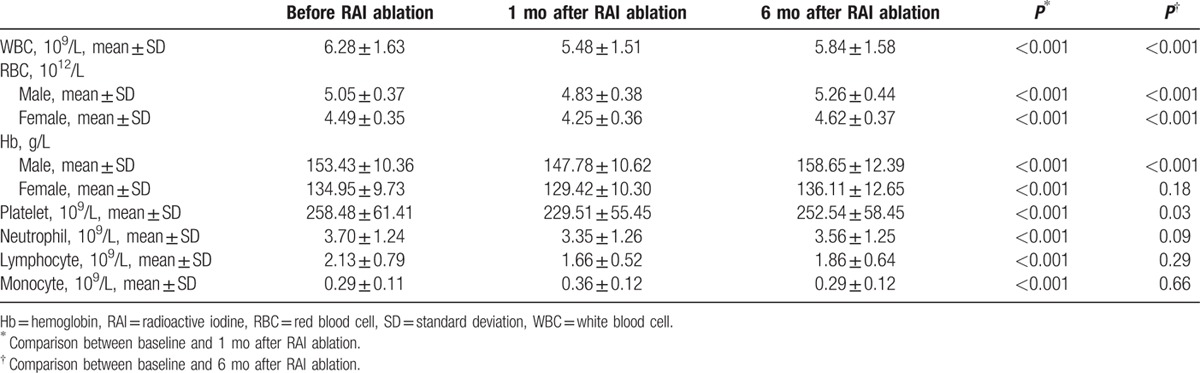
Complete blood cell changes 1 month and 6 month after RAI treatment from baseline.

## 4 Discussion

We found that RAI ablation with administered activities approximating 3700 MBq was associated with significant declines of WBC, platelet, and lymphocyte that persisted for at least 6 months after ablation. However, the magnitudes of the declines were without clinical significance. Hematologic toxicity is a common side effect of RAI treatment.^[7,13]^ Few studies described the effects of a single-dose RAI on CBC. We were able to make this clinical observation because of the relatively large number of patients with DTC treated at our institution with regular hematologic monitoring. Even though mild changes in the CBC were not associated with adverse clinical outcomes, significant decline in WBC, platelet, and lymphocyte was a poignant reminder that RAI ablation could be associated with mild bone marrow suppression for up to 6 months.

Several studies have demonstrated that RAI therapy, especially repeated high-dose RAI treatments, can be associated with leukopenia, thrombopenia, and anemia,^[7,10,14–16]^ and even an increased risk of leukemia.^[3,4]^ For instance, the study of Haynie and Beierwaltes^[10]^ demonstrated a transient anemia in 35% with a mean cumulative administered activity of 9509 MBq, transient leukopenia in 10% with a mean cumulative activity of 12,284 MBq, and thrombopenia in 3% of the patients with a mean cumulative activity of 17,316 MBq. WBC and platelet counts normalized in all patients 1 year after RAI therapy, but anemia persisted in 5% of the patients, which was probably related to the much higher cumulative administered activity received by those patients as compared to a single 3700 MBq RAI activity given to our patients. Molinaro et al^[7]^ evaluated the impact of a single RAI ablation, after traditional THW or recombinant human thyroid stimulating hormone (rhTSH) stimulation, on the CBC obtained 1 year after RAI treatment. They found a significant transient decrease of platelet and leukocyte counts, but they did not discuss the gender differences on CBC changes. Padovani et al^[16]^ showed a significant decrease of blood cell counts in patients with DTC (62% with distant metastases at presentation) after repeated high-dose RAI treatments. Prinsen et al^[15]^ also showed platelet and leukocyte were transiently decreased after repeated RAI therapies in a DTC population that was represented by patients with abnormal blood counts.

RAI therapy requires a status of hypothyroidism, or a TSH level of at least 30 mIU/L.^[2]^ RAI is primarily cleared from the body by renal filtration, and total-body RAI clearance can be reduced by 25% to 30% in patients with hypothyroidism.^[17–19]^ Estimated by cockcroft-gault formula, the majority of our cohort of DTC patients’ creatinine clearance (112.57 ± 32.80) was among normal reference. Only 27 of 385 patients’ creatinine clearance were below 75 mL/min (according to KDIGO Clinical Practice Guideline, mild chronic renal insufficiency is defined as creatinine clearance <75 mL/min).^[20]^ So we consider the impact of THW on renal function is generally small. But this issue is important for such investigation, which should be taken into consideration for our future studies. Since RAI is cleared from the blood and body more rapidly after rhTSH preparation than traditional THW.^[21,22]^ Some reports demonstrated that the dose-related side effects of RAI may be less likely to occur with the use of rhTSH. Rosario et al^[23]^ demonstrated that the mean decrease percentage of platelet within the first 2 months after 3700 MBq RAI ablation was higher in patients prepared with THW than those prepared with rhTSH (45% vs 20%, *P* < 0.01). However, a recent study reported that the changes in total WBC and platelets obtained 1 year after RAI treatment were not related to the method of preparation or the administered activity of RAI.^[7]^ The use of rhTSH for the therapy of metastatic DTC is still controversial.^[24]^ Some studies show that rhTSH preparation is just suitable for young patients with a limited extent of disease, who have an overall good prognosis and a long disease-free survival.^[25–27]^ What is more, the cost of rhTSH preparation is much more expensive than THW preparation. Besides, rhTSH has not been introduced in China.

Surprisingly, we found a significant increase of RBC and Hb in both male and female 6 months after treatment. A possible explanation for this phenomenon could be that the short-term hypothyroid state prior to the RAI treatment may have caused a relatively anemic state, which recovers after the administration of thyroid hormone after RAI therapy.^[2,28]^ Furthermore, the postsurgical state of patients could have caused a lowered Hb level due to surgery-related blood loss. It is thus possible that the baseline values of RBC and Hb in males and females were relatively low. Relative sparing of RBC production after radiation is also a common phenomenon,^[7,29]^ which could explain why decline of Hb was observed 1 month post-treatment in our study. At the same time, some studies reported hormesis effect on hematopoietic system induced by low-dose radiation may be related to the increasing of cytokines.^[30]^ This effect may also give another possible explanation to the findings of the present study.

Ionizing radiation dose-dependent declines in hematologic cells have been documented.^[31]^ Declines of lymphocyte and granulocyte counts happen over hours, days, and weeks, respectively. Decline of platelet count occurs over a period of days, consistent with their half-life. Just like the development of salivary gland side effects,^[6,7]^ there appears to be much individualized variability in the susceptibility to having a decline in WBC, platelet, and lymphocyte, or a rise in RBC and Hb in response to RAI exposure. Either the threshold for developing alterations in the CBC differs among patients or factors other than administered activity are having a significant impact on the actual bone marrow dose. Alternatively, differences in patients’ ability to repair radiation-induced damage could also contribute to differences in susceptibility of an individual to develop CBC abnormalities in response to RAI radiation. This retrospective study cannot differentiate the cause of the individual dose–response variations. While no significant dose–response relationship was detected in this study, we found that the dose of ablation therapy has a significant impact on platelet. The <3700 MBq group mostly consist of those low-risk DTC patients with little to no residual functioning thyroid tissue in the neck or evidence of significant metastatic disease. The more than 4440 MBq group primarily compose of those high-risk DTC patients with metastases and have a higher radiation burden. This is partly proved that patients with high RAI uptake in their metastatic lesions could be exposed to a larger administered activity in the bone marrow with more subsequent damage of the blood cell lineages.

In addition, another issue relating radiation needs a piece of comment. Many participants in our study cohort tended to worry about the radiation safety of RAI, even after our repeated explanations about the RAI safeness if they follow our protocols. They thought that ionizing radiation could be harmful to their health, particularly the blood cells. For removing their fears, if patients wanted to check their CBC at the follow-up 1 month after RAI therapy in our hospital, we would oblige to perform CBC test. This is the main reason that we had 197 of 385 DTC patients’ data available in our CBC records 1 month after RAI therapy.

This study has several limitations. First, our data presented a short-term follow-up (6 months after ablation), we could not determine long-term CBC changes. Second, this study did not include lower dose (around 1110 MBq) for low-risk patients, we plan to continue the study with wide range of RAI doses in the future. Third, rhTSH is not available in China, so we cannot compare the difference between rhTSH and THW. Forth, we did not consider the impact of renal function of patients with hypothyroidism on bone marrow function. Finally, we did not measured bone marrow dose.

In conclusion, our study showed that after a single RAI administration, WBC and platelet decreased continuously from 1 to 6 months, yet RBC and Hb transiently decreased at 1 month and then increased to levels even higher than baseline. The decline of platelet in females was more obvious than in males at 3700 to 4440 MBq RAI. On the contrary, the rises of RBC and Hb in males were higher than in females. Due to these minor changes were still among normal reference ranges and without clinical significance (requiring immediate medical attention), clinicians should not decrease the use of RAI ablation in moderate- to high-risk DTC patients in whom the benefits of ablation are likely to outweigh these minor risks.
